# A Challenging Case of Giant Gastric Perforation: Insights From a Case Report

**DOI:** 10.7759/cureus.77691

**Published:** 2025-01-20

**Authors:** Mahdi A Abdulrasoul, Hassan A Alqumber, Hussain J Aljubran, Abrar Z Kadhem

**Affiliations:** 1 General Surgery Department, Qatif Central Hospital, Qatif, SAU; 2 Faculty of Medicine, Mansoura University, Mansoura, EGY

**Keywords:** case report, gastrectomy, gastric perforation, gastric ulcer disease, laparoscopy

## Abstract

Gastric ulcer disease is a prevalent condition in the general population. The presence of giant gastric ulcers poses a challenge due to their increased risk of perforation and potential for malignancy. A giant gastric ulcer was discovered in a 43-year-old male with a history of gastric ulcer perforation and subsequent complications, including recurrent bleeding episodes despite multiple interventions. This case highlights the complexities of managing recurrent bleeding in patients with giant gastric ulcers and the need for a multifaceted approach combining medication and surgical intervention. The patient underwent initial treatment, involving exploratory laparotomy and gastrojejunostomy tube placement (which is a device that is inserted into the jejunum to provide nutrition, fluids, and medications), followed by various surgical procedures and was managed successfully. This case highlights the complex management of recurrent bleeding from a giant gastric ulcer, requiring multiple interventions and emphasizing the importance of long-term follow-up to avoid its possible complications.

## Introduction

Gastric ulcer disease is a frequently occurring condition, with a lifetime prevalence of 5-10% and an annual incidence of 0.1-0.3% in the general population [1]. This condition most frequently develops due to acid damage to the lining of the stomach, leading to erosion of the mucosa and exposure of underlying tissues to digestive secretions [1]. Historically, this disease has been associated with a high acid secretion environment, dietary factors, and stress [2]. However, the epidemiology of this disease has been altered by the rising prevalence of *Helicobacter pylori* infection, the widespread use of nonsteroidal anti-inflammatory drugs, and the increase in alcohol and tobacco misuse. Despite significant reductions in incidence, hospitalization rates, and mortality over the past three decades, complications such as perforations still exist [3]. Perforated peptic ulcers account for around 2-14% of all peptic ulcers and are associated with a high morbidity rate of 20-70% and a death rate of 15-40% [4]. The size of the hole significantly impacts the death rate and prognosis of this disease. Perforations smaller than 5 mm result in a 6% mortality rate, whereas perforations between 5 and 10 mm increase the mortality rate to 19%. Meanwhile, perforations larger than 10 mm are associated with a mortality rate of approximately 24% [5].

Giant peptic ulcer perforations present a complex surgical dilemma. While omentopexy is a valuable technique, its effectiveness can be limited to very large defects [6]. In such cases, alternative approaches like jejunal serosal patching or omental plugging may be necessary [7]. Remarkably, managing giant peptic ulcer disease is challenging, due to different factors. Hence, this study highlights the complexities of managing recurrent bleeding from a giant gastric ulcer.

## Case presentation

**Figure 1:**
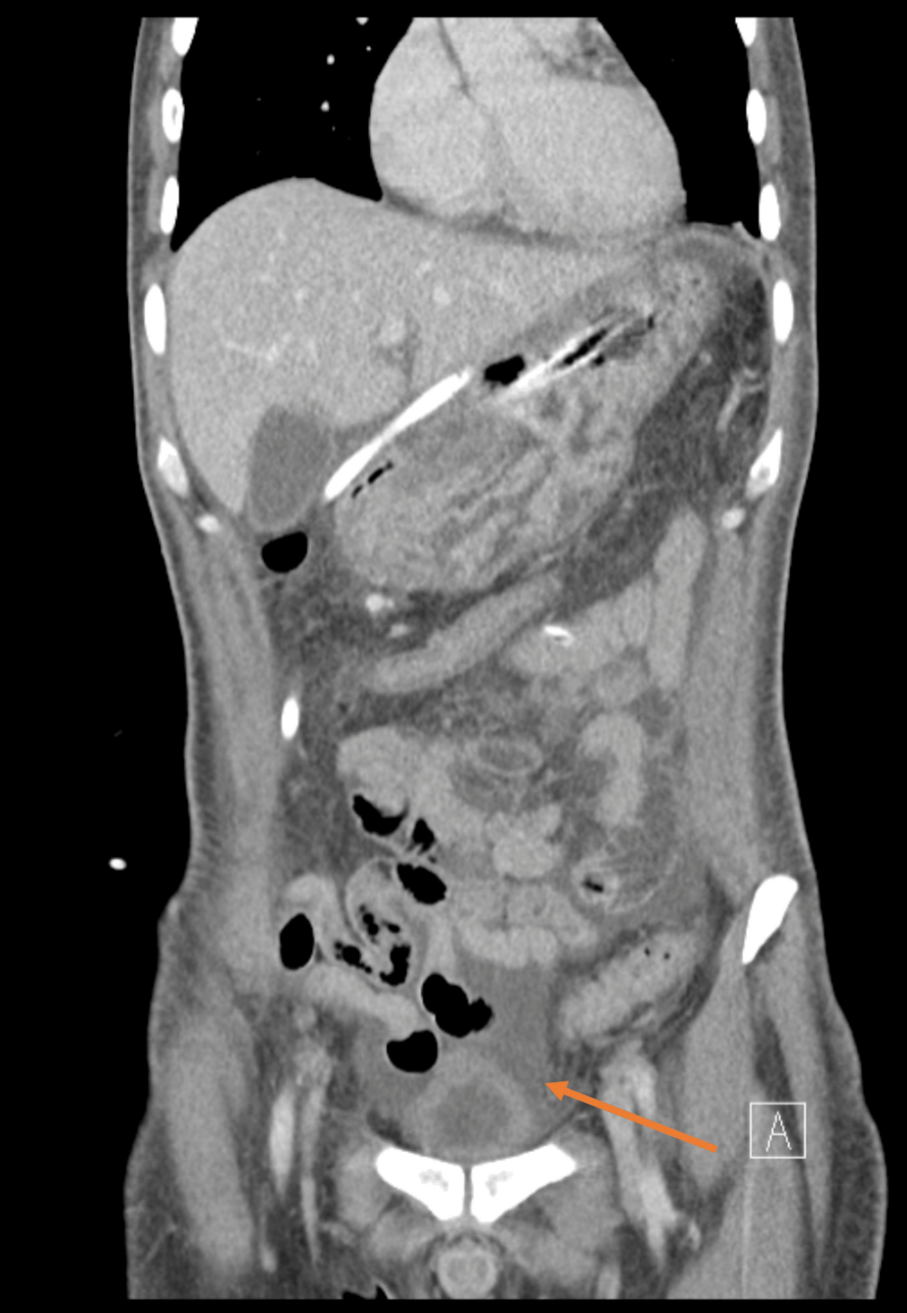
Abdominal computer tomography (coronal scan) with pneumoperitoneum (arrow)

A 43-year-old medically free male, presented to the emergency department complaining of upper GI bleeding with progressive fatigue and dizziness. The patient had a history of gastric ulcer perforation and incisional hernia, both of which were operated on one year ago. There was no other smoking, family, or psychological history. On examination, the patient was vitally stable but appeared pale and fatigued. Abdominal examination revealed a soft and lax abdomen with a jejunostomy tube in place for feeding from his previous operation. However, no signs of peritonitis or active bleeding were noted. His initial laboratory findings were within normal except for a low hemoglobin level of 5.4 g/dL, as seen in Table 1. Accordingly, the patient was admitted and started on omeprazole infusion at a rate of 8 mg per hour intravenously and two units of packed red blood cells for anemia correction. Despite the continuous management of the patient, his hemoglobin level remained low, and the patient experienced recurrent upper GI bleeding episodes. Thus, the patient had a computed tomography (CT) scan within 12 hours of his admission, which showed pneumoperitoneum (Figure 1). Later, the patient underwent damage control surgery aimed at lesion control three days after his admission. Intraoperatively, a perforation was identified at the gastroesophageal junction on the posterior wall. Hence, a primary closure of the perforated area was done and an omentum patch was applied. During that time, supportive care measures were administered, including omeprazole infusion to manage gastric acidity and a jejunostomy feeding regimen to ensure nutritional support.

**Table 1:** Initial laboratory investigations during the first visit.

Tests	Reference range	Patient values
Complete blood count		
White blood cell (WBC)	5-10 x 10^3^/uL	9.61
Hemoglobin	11.5-12.5 g/dL	5.4
Mean corpuscular volume (MCV)	78-96 fL	76.5
Platelet	150-430 x10^3^/uL	294
Coagulation profile		
Prothrombin time (PT)	11.5-15 sec	13.8
Partial thromboplastin time (PTT)	25-33 sec	26.7
International normalized ratio (INR)	0.85-1.15	1.03
Serum biochemistry		
Blood urea nitrogen (BUN)	1.66-8.33 mmol/L	3.3
Serum creatinine	53-106 umol/L	52
Sodium (Na)	135-153 mmol/L	141
Potassium (K)	3.5-5.3 mmol/L	4.08
Calcium (Ca)	2.1-2.55 mmol/L	2.09
Aspartate transaminase (AST)	0-38 U/L	11.7
Alanine transaminase (ALT)	10-50 U/L	10
Albumin	35-52 g/L	36.5
Total protein	64-82 g/L	65.4

During his hospital admission, the patient's condition improved gradually. Furthermore, he underwent an upper GI endoscopy 10 days after his procedure, which showed a 2.5 cm markedly healing giant ulcer in the distal to the gastroesophageal junction at the posterior wall with minimal bleeding. Hence, the patient was discharged after 14 days of his presentation in a stable condition on oral omeprazole, multivitamin supplementation, and jejunostomy feeding, and was scheduled for close follow-up appointments. Nine months after his discharge, the patient presented again to the emergency department with massive upper GI bleeding. Therefore, the patient underwent an emergent partial gastrectomy (PG), achieving definitive control of the bleeding, and was discharged after that in a stable condition with a recommendation to continue his medication and close follow-up appointments. Three years after his operation, the patient presented to the clinic with no active complaint, emphasizing the resolution of his previous issue.

## Discussion

A giant gastric ulcer is characterized by its size, typically exceeding 2 cm in diameter, and is commonly located along the lesser curvature, particularly at the incisura angularis [8]. The risk of perforation in the giant gastric ulcer accounts for 2.125% of the total perforated gastric ulcers [5]. In addition, they may raise suspicion for malignancy, particularly when presenting with scalloped margins and loss of rugal folds surrounding the ulcer [9]. Therefore, this case underscores the complexity and challenges of managing a patient with recurrent bleeding episodes stemming from a history of gastric ulcer perforation and subsequent surgical interventions.

A systematic review comparing the safety and effectiveness of proton pump inhibitor (PPI) and histamine H2 receptor antagonist in treating ulcers or perforation at different sites showed that omeprazole exhibits superior efficacy in ulcer healing and pain relief. Moreover, patients who do not respond to histamine H2 receptor antagonists may benefit from switching to a PPI for better treatment outcomes [10]. In this study, the reported patient experienced recurrence of bleeding, despite being prescribed PPI. The reason of recurrence could be due to a combination of factors, including non-compliance and gastric fragility. Besides PPI, PG emerges as the procedure of choice for achieving both therapeutic objectives of complete ulcer removal and minimizing recurrence. However, PG demands significant technical expertise and longer operating times and may entail blood transfusions [11]. 

Despite these challenges, studies reported lower long-term recurrence rates with PG, albeit with a higher peri-operative mortality rate. Meanwhile, another study revealed that PG and gastrojejunostomy were deemed preferable due to the high risk of malignancy and leak following primary closure in giant gastric ulcers [12]. Alternatively, operative procedures involving drains and feeding jejunostomy tubes offer a safe and reliable option, particularly in critically ill patients or settings with limited technical resources [13]. In our case, the patient also underwent an operative procedure involving drains and feeding jejunostomy as a part of the initial treatment and then underwent PG for ulcer removal after the recurrence of his presentation. In situations where PG is not feasible due to limited technical expertise or infeasibility of omental plugging, options like jejunal serosal patching may be considered [14]. Apparently, these alternative procedures not only extend the duration of surgery but also demand a degree of surgical expertise that may not always be accessible in emergency situations. Moreover, these techniques carry their own risks of morbidity, which can collectively influence the patient’s overall outcome. Most critically, none of these approaches is entirely free from the possibility of postoperative leakage, a key concern when considering the use of an omental patch for larger perforations [15,16]. Although less common, these alternatives provide viable options in challenging clinical scenarios.

## Conclusions

In this study, we highlighted the complex management and challenges associated with recurrent bleeding from a giant gastric ulcer perforation. Our findings underscore the importance of a multidisciplinary approach in addressing such cases, involving timely surgical interventions, appropriate postoperative care, and long-term follow-up to mitigate complications. While our case demonstrates successful management, it also emphasizes the need for further research into optimizing treatment strategies for giant gastric ulcers, particularly in identifying factors that contribute to recurrence despite standard therapies. Future studies should focus on developing advanced therapeutic approaches to improve patient outcomes and explore the role of innovative surgical techniques or adjunctive therapies in managing complex ulcers.

## References

[1] Lanas A, Chan FKL (2017). Peptic ulcer disease. Lancet.

[2] Malmi H, Kautiainen H, Virta LJ, Färkkilä N, Koskenpato J, Färkkilä MA (2014). Incidence and complications of peptic ulcer disease requiring hospitalisation have markedly decreased in Finland. Aliment Pharmacol Ther.

[3] Sonnenberg A (2007). Time trends of ulcer mortality in Europe. Gastroenterology.

[4] Chung KT, Shelat VG (2017). Perforated peptic ulcer - an update. World J Gastrointest Surg.

[5] Ali WM, Ansari MM, Rizvi SA, Rabb AZ, Mansoor T, Harris SH, Akhtar MS (2018). Ten-year experience of managing giant duodenal ulcer perforations with triple tube ostomy at tertiary hospital of North India. Indian J Surg.

[6] Bekele A, Kassa S, Taye M (2016). The jejunal serosal patch procedure: a successful technique for managing difficult peptic ulcer perforation. East Cent Afr J Surg.

[7] Elheny A, Shehata AM, Saleh AF, Sageer EE (2015). Duodenal injuries: how to deal with it?. Egypt J Surg.

[8] Tarnawski AS, Ahluwalia A (2021). The critical role of growth factors in gastric ulcer healing: the cellular and molecular mechanisms and potential clinical implications. Cells.

[9] Zhao Q, Chi T (2021). Biopsy in emergency gastroscopy does not increase the risk of rebleeding in patients with Forrest I acute nonvariceal upper gastrointestinal bleeding combined with suspected malignant gastric ulcer: a multicenter retrospective cohort study. BMC Gastroenterol.

[10] Begg M, Tarhuni M, N Fotso M (2023). Comparing the safety and efficacy of proton pump inhibitors and histamine-2 receptor antagonists in the management of patients with peptic ulcer disease: a systematic review. Cureus.

[11] Croagh D, Michalski CW, van Berge Henegouwen MI, Alfieri S (2023). Diagnosis and management of pancreatic insufficiency in patients with gastrectomy due to cancer or gastric ulcers: a virtual roundtable expert discussion. Expert Rev Gastroenterol Hepatol.

[12] Kumar P, Khan HM, Hasanrabba S (2014). Treatment of perforated giant gastric ulcer in an emergency setting. World J Gastrointest Surg.

[13] Waliye HE, Wright GP, McCarthy C, Johnson J, Scales A, Wolf A, Chung M (2017). Utility of feeding jejunostomy tubes in pancreaticoduodenectomy. Am J Surg.

[14] Lorange M, Smeak DD (2020). Comparison of a simple continuous versus simple interrupted suture pattern for the repair of a large, open duodenal defect with a jejunal serosal patch in a canine cadaveric model. Am J Vet Res.

[15] Chaudhary A, Bose SM, Gupta NM, Wig JD, Khanna SK (1991). Giant perforations of duodenal ulcer. Indian J Gastroenterol.

[16] Karanjia ND, Shanahan DJ, Knight MJ (1993). Omental patching of a large perforated duodenal ulcer: a new method. Br J Surg.

